# DCUN1D3 activates SCF^SKP2^ ubiquitin E3 ligase activity and cell cycle progression under UV damage

**DOI:** 10.18632/oncotarget.11302

**Published:** 2016-08-16

**Authors:** Shuai Zhang, Jing Huang, Taiping Shi, Fanlei Hu, Li Zhang, Ping-Kun Zhou, Dalong Ma, Teng Ma, Xiaoyan Qiu

**Affiliations:** ^1^ Department of Immunology, School of Basic Medical Sciences, Peking University, Beijing, China; ^2^ Chinese National Human Genome Center, Beijing, China; ^3^ Department of Radiation Toxicology and Oncology, Beijing Key Laboratory for Radiobiology (BKLRB), Beijing Institute of Radiation Medicine, Beijing, P. R. China

**Keywords:** DCUN1D3, CAND1, SCF^SKP2^, p27, cell cycle

## Abstract

Our previous study showed that knockdown the endogenous expression of *DCUN1D3* (also called *SCCRO3* or *DCNL3*) blocked the S phase progression after UV irradiation. Here, we show that the silence of *DCUN1D3* can increase the cyclin-dependent kinase inhibitor p27 protein levels after UV irradiation. Through Co-immunoprecipitation experiments, we found that DCUN1D3 bound to CAND1. And DCUN1D3 knockdown synergized with CAND1 over-expression in arresting the S phase. Given the CAND1′s established role in Cullin-1 neddylation, we found Cullin-1 was less neddylated in DCUN1D3 deficient cells. So the silence of DCUN1D3 can inhibit the formation of SCF^SKP2^ complex by reducing Cullin-1 neddylation. Given that p27 is the primary target of SCF^SKP2^ complex, the cells lost DCUN1D3 showed a remarkable accumulation of p27 to cause S phase block.

## INTRODUCTION

In previous study, we identified a novel UV-responsive protein DCUN1D3, which is involved in UV-related cell cycle check point, cell growth and cell survival [1]. We found that UV can increase DCUN1D3 expression on both mRNA and protein level. Knockdown endogenous expression of DCUN1D3 blocked the S phase progression after UV irradiation [1]. DCUN1D3 protein belongs to the DCNLs family, which have been implicated in the regulation of activity of SCF ligases [2–7]. However, the detailed role of DCUN1D3 in cell cycle regulation remains to be elucidated.

In mammals, p27 is ubiquitinated by the SCF^SKP2^ ubiquitin E3 ligase. In particular, SKP2, an F box protein, binds and targets p27 for polyubiquitination and subsequent proteolysis [8, 9]. Cullin-1 (CUL1) is an essential component of the SCF (SKP1, CUL1/CDC53, Rbx1 and F box proteins) ubiquitin E3 ligase complex that controls the protein levels of many regulatory proteins such as β-catenin, IκB, and p27^KIP1^ [10, 11]. CUL1 binds to SKP1, which in turn binds with the substrate-targeting subunit, the F box proteins [11, 12]. CUL1 also associates with the RING finger protein ROC1 (also called RBX1 or HRT1) which links SCF to the ubiquitin-conjugating enzyme E2 and the activating enzyme E1 for the ubiquitin transfer reaction [11, 13].

Cullin-Ring type E3 ubiquitin ligases are activated through a process called neddylation where the CUL1 component is covalently modified by ubiquitin-like protein NEDD8. Like ubiquitination, neddylation of CUL1 is activated through heterodimer E1-activating enzyme (APPBP1/UBA3) and E2-conjugating (UBE2M and UBE2F) enzymes [14]. This process is essential for the ubiquitination ligase activity of SCF complexes and is required for ubiquitin-dependent proteolysis of p27 [15, 16]. Conversely, deneddylation of CUL1 has been shown to cleave NEDD8 from CUL1 [17]. Both *in vitro* and *in vivo*, CAND1 is an inhibitor for SCF^SKP2^ complex through binding to unneddylated CUL1 [18, 19]. Biochemical and crystal structural studies of CAND1-CUL1 complexes suggest that the binding site for SKP1 and SKP2 is at the N-terminal region of CUL1 and that this site is blocked when CAND1 is bound to CUL1. Neddylation of CUL1 at a critical C-terminal lysine residue, however, prevents CUL1 from binding to CAND1, thus allowing CUL1 to be able to bind to SKP1 and SKP2 to form the SCF^SKP2^ complex [20, 21, 22].

Through bioinformatics analysis and a series of Glutathione S-Transferase (GST) Pull-down screen in mouse cell lines, mouse DCUN1D3 was recently implicated as a novel binding partner of CAND1 and unneddylated CUL1. However, there has yet to be no further functional investigation. In this study, we demonstrate that human DCUN1D3 binds with CAND1 and CUL1 in cultured human cell line. After UV damage, CAND1 overexpression can augment DCUN1D3 inhibition-dependent S phase arrest. Depletion of DCUN1D3 expression increased p27 protein level. Therefore, DCUN1D3 activates the SCF E3 ligase and exerts its function in the cell cycle progression after UV damage.

## RESULTS

### DCUN1D3 binds with CAND1

We identified a novel UV-responsive protein DCUN1D3, which activates the UV-related cell cycle checkpoint. As a step towards understanding the molecular function of DCUN1D3, we sought to identify its interacting proteins. We used a Bayesian framework in PrePPI database (http://bhapp.c2b2.columbia.edu/PrePPI) to predict protein-protein interactions by combining structural modeling, functional, evolutionary and expression information [23, 24]. Besides Rbx1 and UBE2M, CAND1 was predicted to interact with DCUN1D3. An interaction network model is shown in Supplementary Figure S1A. A complete list of predicted interaction proteins are shown in Supplementary Table S1.

**Figure 1:**
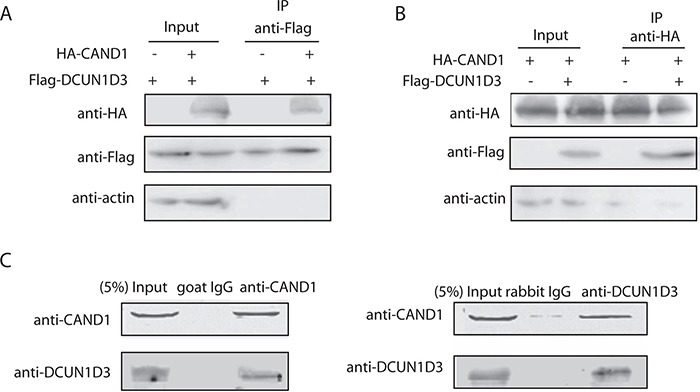
DCUN1D3 binds with CAND1 **A.** Coimmunoprecipitation of CAND1 and DCUN1D3 in HeLa cells. Cells were cotransfected with HA-CAND1 and Flag-DCUN1D3 plasmids or cotransfected with HA-vector and Flag-DCUN1D3 plasmids. Total cell extracts were subjected to IP using anti-Flag as indicated. Immunoprecipitated proteins were then analyzed for the presence of HA-CAND1 by Western blot. **B.** Cells were cotransfected with HA-CAND1 and Flag-DCUN1D3 plasmids or cotransfected with HA-CAND1 and Flag-vector control plasmids, then cell extracts were immunoprecipitated with an anti-HA antibody and immunoblotted with an anti-Flag antibody. **C.** Endogenous CAND1 binds with DCUN1D3 in HeLa cells. Total cell extracts were subjected to IP using either an anti-CAND1 or an control IgG as indicated. Immunoprecipitated proteins were then analyzed for the presence of DCUN1D3 by Western blot. In the right panel, except that cell extracts were immunoprecipitated with an anti-DCUN1D3 antibody and immunoblotted with an anti-CAND1 antibody.

To confirm the association of DCUN1D3 and CAND1 in mammalian cells, Flag-DCUN1D3 and HA-CAND1 plasmids were cotransfected into HeLa cells. Cell lysates were subjected to IP with an anti-Flag antibody. Western blot analysis revealed that Flag-DCUN1D3 co-precipitated with HA-CAND1 (Figure 1A). Reciprocally, Flag-DCUN1D3 was detected in the HA-CAND1 immunoprecipitates, demonstrating that the two proteins existed as a complex in mammalian cells (Figure 1B). A sturctural docking model of DCUN1D3-CAND1 interaction was also generated by Frodock 2.0 (J.I. Garzón et al., 2009) in Supplementary Figure S1B.

Furthermore, we sought to examine whether interaction occurs between endogenous DCUN1D3 and CAND1. Endogenous CoIP analysis provided evidence for the endogenous DCUN1D3-CAND1 interaction. This interaction was specific as shown in Figure 1C. The interaction between DCUN1D3 and CAND1 was also observed by using a pull-down approach (Supplementary Figure S1C). These findings suggest that DCUN1D3 binds with CAND1.

### Combination of DCUN1D3 knockdown and CAND1 overexpression augments S phase arrest after UV irradiation

**Figure 2:**
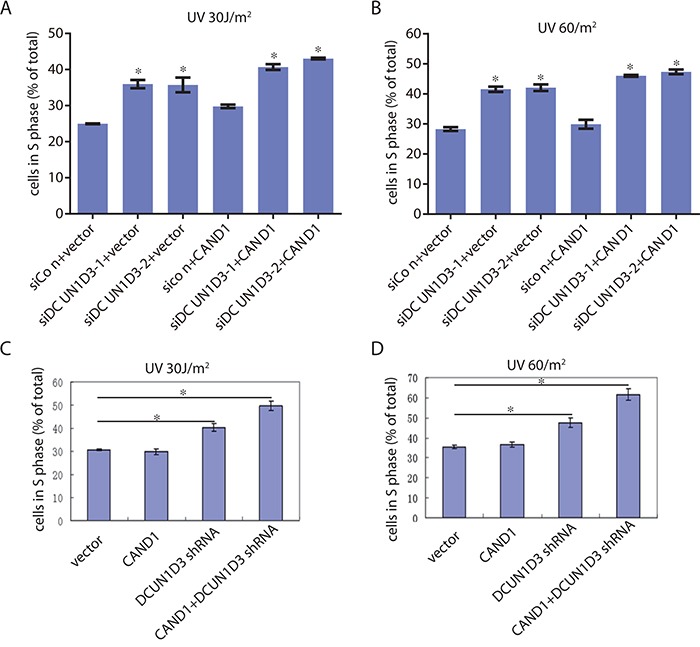
The synergistic effects of DCUN1D3 silencing and CAND1 overexpression on the cell cycle profile of HeLa cells **A.** and **B.** HeLa cells were transfected with DCUN1D3 siRNA combined either with vector or HA-CAND1, then treated with 30 J/m^2^ UV or 60 J/m^2^ UV. 48h after treatment, cells were collected and analyzed for cell cycle profile. The percentage of cells in S phase (% of total) were shown in bar graph. **C.** and **D.** HeLa cells were transfected with HA-CAND1, DCUN1D3 shRNA solely or combined with HA-CAND1, then treated with 30 J/m^2^ UV or 60 J/m^2^ UV. 48h after treatment, cells were collected and analyzed for cell cycle profile. The percentage of cells in S phase (% of total) were shown in bar graph.

As we know, knockdown the endogenous DCUN1D3 can block S phase after UV irradiation. Then we asked whether CAND1 can influence DCUN1D3-related cell cycle transition. To examine this possibility, two validated siRNAs from the previous study [1] were co-transfected with CAND1, cells were irradiated with 30J and 60J UV respectively, and then harvested for DNA content analysis by FACS. The results indicated that under UV irradiation condition, the DCUN1D3 siRNA transfected cells showed a 15 ± 5% increase of cells in S phase than the control cells 48h after 30J and 60J UV irradiation respectively, co-transfection of DCUN1D3 siRNA and CAND1 in HeLa cells showed a 20 ± 2% increase of cells in S phase than the control cells 48h after 30J and 60J UV irradiation respectively (Figure 2A and 2B). There was no significant difference between CAND1 and vector group either under 30J or 60J UV irradiation.

Furthermore, one DCUN1D3 candidate shRNA and CAND1 were co-transfected into HeLa cell lines under same UV-treated condition and the cells were harvested for cell cycle profile. The results indicated that under UV irradiation condition, the DCUN1D3 shRNA transfected cells showed a 10 ± 2% and 15± 3% increase of cells in S phase than the control cells 48h after 30J and 60J UV irradiation respectively, the DCUN1D3 shRNA transfected with CAND1 in HeLa cells showed a 20 ± 2% and 24 ± 3% increase of cells in S phase than the control cells 48h after 30J and 60J UV irradiation respectively (Figure 2C and 2D). There was no significant difference between CAND1 and control group either under 30J or 60J UV irradiation (Figure 2C and 2D). DCUN1D3 protein levels after DCUN1D3 shRNA transfection were significantly decreased at 48, 72, 96h in HeLa (Supplementary Figure S2).

Therefore, these results suggest that S-phase progression after UV in the DCUN1D3 shRNA transfected group was blocked significantly and CAND1 can augment this S phase block induced by DCUN1D3 knockdown.

### Inhibition of the endogenous DCUN1D3 expression can increase the expression of p27 and inhibit the formation of SCF^SKP2^ ubiquitin E3 ligase complex after UV irradiation

**Figure 3:**
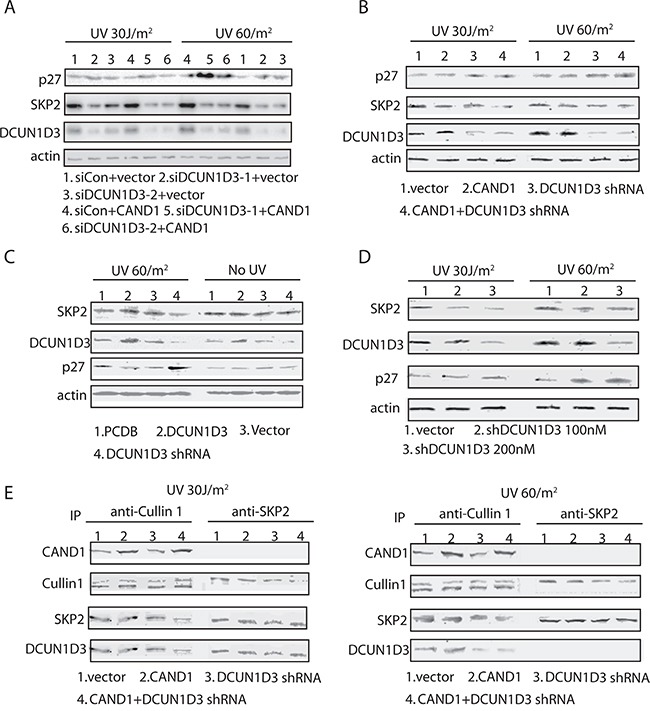
DCUN1D3 supresses the expression of p27 and SKP2 in HeLa cells and the formation of SCF^SKP2^ ubiquitin E3 ligase complex in UV-treated HeLa cells **A.** HeLa cells were co-transfected DCUN1D3 specific siRNAs and control vector or CAND1 over-expression plasmids. Cells were treated 30 J/m^2^ UV or 60 J/m^2^ UV, then cell lysates were prepared 48h after the UV treatment and subjected to Western blot with the corresponding antibodies indicated. **B.** HeLa cells were transfected with the indicated combinations of plasmids expressing control shRNA, DCUN1D3 specific shRNA, CAND1 and its empty vectors. Cell lysates were prepared 48h after the UV treatment and subjected to Western blot with the corresponding antibodies indicated. **C.** HeLa cells were transfected with PCDB, PCDB-DCUN1D3, control shRNA, DCUN1D3 specific shRNA. Cell lysates were prepared 48h after the UV treatment or not and subjected to Western blot with the corresponding antibodies indicated. **D.** Hela cells were transfected with DCUN1D3 control shRNA and DCUN1D3 specific shRNA (100nM and 200nM). 48h after UV treatment, cells were harvested and analyzed by Western blot with the indicated antibodies. **E.** Cells were treated the same as in (A). And cells were harvested at 48h after UV treatment. Cell lysates were processed for immunoprecipitation with indicated antibodies and analyzed by SDS-PAGE and Western blot with the antibodies indicated.

To further dissect the mechanisms of DCUN1D3-activated S cell cycle arrest after UV irradiation, we examined expression levels of cell cycle related proteins. Using DCUN1D3 siRNAs and shRNA, we have inhibited the DCUN1D3 expression in HeLa cells. We found that inhibiting the expression of DCUN1D3 led to a marked increase in p27 levels and a substantial decrease in SKP2 expression after UV irradiation (Figure 3A and 3B). And DCUN1D3 siRNAs and shRNA co-transfected with CAND1 in HeLa cells can augment p27 increase and SKP2 decrease after UV irraditation (Figure 3A and 3B). But there was no significant p27 or SKP2 change without UV treatment (Figure 3C). Moreover, we also checked whether p27 mRNA level changed after DCUN1D3 knockdown and/or CAND1 over-expression, however, as it showed in Supplementary Figure S3, we found no significant difference between various groups.

To more broadly examine the role of DCUN1D3 in regulation of p27 levels, we also silenced the DCUN1D3 with 100nM and 200nM shRNA in HeLa cells under 30J and 60J UV irradiation. As shown in Figure 3C, silencing of DCUN1D3 under 30J and 60J UV irradiation caused a potent accumulation of p27, while SKP2 levels decreased (Figure 3D).

Since the major components of the SCF^SKP2^ complex appear to be present at comparable levels, we wondered if assembly of the SCF^SKP2^ complex could be inhibited. To address this question, we isolated CUL1 or SKP2 complexes from HeLa cells treated with DCUN1D3 shRNA or CAND1 or both after UV irradiation. The immunoprecipitates were analyzed by SDS-PAGE and Western blotting for the presence of associated proteins. From these experiments, we found that less SKP2 were associated with CUL1 complexes recovered from DCUN1D3 shRNA or DCUN1D3 shRNA and CAND1 both treated cells than from control cells (Figure 3E). Reciprocally, less CUL1 is present in SKP2 complexes from cells transfected with DCUN1D3 shRNA or DCUN1D3 shRNA and CAND1 than from control cells (Figure 3E). The data suggest that reduction of DCUN1D3 levels caused a defect in the association of SKP2 with CUL1 after UV irradiation.

### DCUN1D3 binds to CUL1 and knockdown endogenous expression of DCUN1D3 can reduce cullin1 neddylation after UV irradiation

Neddylation of CUL1 promotes the binding of SKP2 to CUL1. So assembly of SCF complex requires neddylation of CUL1 [20]. Because knockdown endogenous expression of DCUN1D3 can cause a defect in the association of SKP2 with CUL1 after UV irradiation. It seemed to be that decreased association of SKP2 and CUL1 upon reduce neddylation of CUL1 may be caused by inhibition of the endogenous DCUN1D3 expression. Indeed, this was found to be the case.

**Figure 4:**
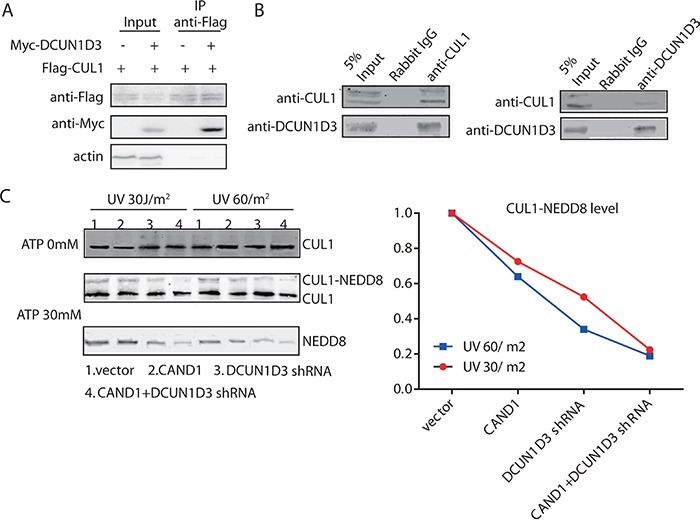
DCUN1D3 binds with CUL1 and stimulates CUL1 neddylation **A.** Coimmunoprecipitation of CUL1 and DCUN1D3 in HeLa cells. Cells were cotransfected with Flag-CUL1 and myc-DCUN1D3 plasmids or cotransfected with Flag-CUL1 and myc-vector plasmids. Total cell extracts were subjected to IP using either an anti-Flag, as indicated. Immunoprecipitated proteins were then analyzed for the presence of myc-DCUN1D3 by Western blot. **B.** Endogenous CUL1 binds with DCUN1D3 in HeLa cells. Total cell extracts were subjected to IP using either an anti-CUL1 or an irrelevant control IgG, as indicated. Immunoprecipitated proteins were then analyzed for the presence of DCUN1D3 by Western blot. In the right panel, except that cell extracts were immunoprecipitated with an anti-DCUN1D3 antibody and immunoblotted with an anti-CUL1 antibody. **C.** The neddylation state of CUL1 is activated by DCUN1D3. HeLa cells were transfected with DCUN1D3 shRNA control vector and pCMV5 HA vectors, DCUN1D3 shRNA control vector and pCMV5 HA-CAND1 vectors, DCUN1D3 specific shRNA vector and pCMV5 HA vectors, DCUN1D3 specific shRNA vector and pCMV5 HA-CAND1 vectors respectively. Cell extracts were prepared 48h after UV treatment and incubated with 0 mM and 30 mM ATP at 30°C for 1 hour. CUL1 complexes were recovered by immunoprecipitation with anti-CUL1 antibody and analyzed by SDS-PAGE and Western blot with the indicated antibodies. The upper band in the CUL1 blot corresponds to neddylated CUL1, as confirmed by reblotting the membrane with an anti-NEDD8 antibody. The band intensity of CUL1-NEDD8 was analyzed by Image J software and summarized in the right gragh.

Given the established role of CAND1 in neddylation, we first assessed that DCUN1D3 bound to CUL1. Myc-DCUN1D3 and Flag-CUL1 plasmids were cotransfected in HeLa cells. Cell lysates were subjected to IP with an anti-Flag antibody. Western blot analysis revealed that myc-DCUN1D3 co-precipitated with Flag-CUL1 (Figure 4A). Furthermore, endogenous CoIP analysis provided evidences for the endogenous DCUN1D3-CUL1 interaction (Figure 4B). The interaction between DCUN1D3 and CUL1 was also observed by using a pull-down approach (Supplementary Figure S4). These findings confirmed that the observed interactions occur *in vivo*. By CoIP experiments, we also found that DCUN1D3 did not bind to SKP2 (data not shown), suggesting that it possibly binds the specific components of the neddylation pathway.

We next assayed if the HeLa cell exracts prepared from cells transfected with CAND1 or DCUN1D3 shRNA or both have a reduced CUL1 neddylation level after UV irradiation. Like ubiquitination, neddylation is an ATP-dependent process. Using an *in vitro* assay which was previously established, at 48h after 30J and 60J UV irradiation, we got the extracts and added 30mM ATP to aliquots of the extracts prior to incubation at 30°C for 1 hour. CUL1 complexes were then immunoprecipitated overnight using an anti-CUL1 antibody, followed by SDS-PAGE and Western blot analysis. Both neddylated and unneddylated CUL1 were detected by the anti-CUL1 antibody. The neddylated CUL1 which migrated slower than the unneddylated CUL1, was produced in an ATP-dependent manner in the extracts and was also recognized by an anti-NEDD8 antibody on Western blot analysis. We found that extracts prepared from cells transfected with DCUN1D3 shRNA were less potent in the *in vitro* cullin1 neddylation reaction than extracts from control cells after UV irradiation (Figure 4C). The extracts prepared from cells cotransfected with DCUN1D3 shRNA and CAND1 were further less potent in the *in vitro* CUL1 neddylation reaction (Figure 4C). The CUL1-NEDD8 levels were also analyzed by Image J and summarized in the line gragh.

## DISCUSSION

In previous study, we have demonstrated that knockdown endogenous expression of DCUN1D3 blocked the S phase progression after UV irradiation [1]. We show that the silence of DCUN1D3 can increase the cyclin-dependent kinase inhibitor p27 protein levels in HeLa cell line after UV irradiation.

It has been well *acknowledged* that p27 is a tumor suppressor, not only because of its activity as a CKI, but also because of evidence from mouse models. In mouse models, p27 functions as a haploinsufficient tumor suppressor and in human cancer, a low level of p27 is correlative with higher tumor grade and poor survival [25, 26, 27]. It is also well established that p27 specifically inhibits the activity of Cdk2 in G1-to-S preventing prmature onset of DNA replication [18]. So the accumulation of p27 can cause the G1-to-S phase block in cells. In our finding, inhibition of the endogenous DCUN1D3 caused the accumulation of p27 and a significant block of the S phase progression under UV damage. So this result is well concerted with the p27's role in cell cycle progression.

p27 is the primary target of SCF^SKP2^ complex. And SKP2 was originally discovered as a protein that associates with cyclin A-CDK2 in transformed cells, and is now known to be an F-box protein of the SCF complex. In conjunction with SKP2, the SCF complex targets p27 CKIs for degradation [28, 29, 30]. However, in the time since it became widely *acknowledged* that SKP2 mediates p27 degradation in G1 phase, several discrepancies have emerged. First, SKP2 is not expressed until early S phase, unequivocally later than the degradation of p27 apparent at G1 [31]. Second, p27 is exported from the nucleus to the cytoplasm at G1, whereas SKP2 is restricted to the nucleus. Third, the downregulation of p27 at the G0-G1 transition occurs normally in SKP2^−/−^ cells and is sensitive to proteasome inhibitors [29]. These temporal, spatial and genetic discrepancies indicate that p27 is degraded at G1 in the cytoplasm by a proteasome-dependent, but SKP2-independent, mechanism. Recently, an E3 enzyme, designated KIP1 ubiquitylation-promoting complex (KPC), that binds with and ubiquitylates p27 in G1 phase and is localized to the cytoplasm of mammalian cells [32]. KPC consists of two subunits, KPC1 and KPC2. KPC1 contains a RING-finger domain near its C terminus, and functions as the catalytic subunit [33]. KPC2 is a member of the UBL-UBA family of proteins. KPC2 stabilizes KPC1, recruits polyubiquitylated p27 and binds with the 26S proteasome, thereby promoting the degradation of p27 [34]. Inhibition of either KPC1 or KPC2 by RNA interference or with dominant-negative mutants delays p27 degradation at the G0-G1 transition. These results indicate that p27 is degraded by two distinct mechanisms: translocation-coupled cytoplasmic ubiquitylation by KPC at the G0-G1 transition and nuclear ubiquitylation by SKP2 during S and G2 phases. Given that, in a subset of breast cancers (32 out of 84 samples) [35], p27 levels were low despite SKP2 not being expressed, it remains possible that the expression of KPC might be elevated in such cases. This dynamic degradation pattern of p27 is also in agreement with our previous findings of the dynamic subcellular localization of DCUN1D3. Since the DCUN1D3 protein is translocated from the cytoplasm to nucleus after UV damage, it is possible that DCUN1D3 participates the SKP2-mediated proteolysis of p27 in nucleus. In light of the cytoplasmic degradation of p27, more experiments are required to elucidate the role of DCUN1D3 in cytoplasm or whether DCUN1D3 is associated with the KPC complex.

Through the CoIP experiments, we found DCUN1D3 bound to CAND1, which accords with the previous report [36]. And CAND1 can augment the S phase block induced by inhibition of DCUN1D3. CAND1 only binds with unneddylated CUL1 and this interaction inhibits the SCF formation, it is possible that lack of CUL1 neddylation promotes the binding of CAND1 to CUL1, thereby preventing the association of F box proteins with CUL1 and causing the inhibition of p27 polyubiquitination [20]. Given the CAND1 established role in neddylation, we have found CUL1 is less neddylated in cells that have lost DCUN1D3. We present evidence that DCUN1D3 regulate the neddylation state of CUL1. Though Huang et al. reported DCUN1D3 antagonizes SCCRO mediated neddylation, we analyzed neddylation in UV treated conditions. It is possible that the competition between DCUN1D3 and SCCRO mediated neddylation *in vivo* is broken after UV damage, which requires further exploration. The DCUN1D3 and SCCRO effects on cul1 neddylation may also be cell cycle dependent.

Neddylation is required for the ubiquitin E3 ligase activities of CUL1 complex. Neddylation activity has also been shown to be required for *in vitro* degradation of p27 in cell extracts, likely through the activation of SCF^SKP2^ activity [8]. In our report, we have shown that when DCUN1D3 is silenced, SCF^SKP2^ complex do not form and thus caused the accumulation of p27.

In this study, we first report the role of human DCUN1D3 in regulation the SCF ubiquitin ligase activity *in vitro* and *in vivo*. It will provide profound knowledge to clarify the roles of DCN1-like protein family (including the DCUN1D3) in cullins neddylation, and further their roles in cell cycle regulation and carcinogenesis.

## MATERIALS AND METHODS

### Plasmids, siRNAs, shRNA, and antibodies

The pcDNA3-Flag-DCUN1D3 and pcDNA3-DCUN1D3-myc plasmids used have been described previously [1]. The pCMV5- Flag-CUL1 and pCMV5- HA-CAND1 vectors were kindly provided by Dr. Y. Rananathan. DCUN1D3 siRNAs were described previously [1]. DCUN1D3 shRNA and the control shRNA were synthesized by GeneChem Corporation (Shanghai, China); the sequences of the various shRNA have been reported previously. The mouse anti Flag, mouse anti myc, and mouse anti actin monoclonal antibodies were purchased from Sigma (St. Louis, MO). The anti-NEDD8, anti-CAND1, anti-SKP2 and anti-p27 antibodies were from Cell Signaling Technology (Beverly, MA). The rabbit anti-DCUN1D3 polyclonal antibody has been described previously. IRDye 800–conjugated or HRP-conjugated secondary antibodies against mouse, rabbit, and goat IgG were purchased from Li-Cor Bioscience (Lincoln, NE).

### Cell culture, transfection, and treatment

HeLa cells were was cultured in Dulbecco's modified Eagle's medium, supplemented with 10% fetal bovine serum. HeLa cells were transfected by electroporation or lipofectamine 2000, as described previously. UV irradiation (30 J/m^2^; 60 J/m^2^) was produced by using a CX-2000 UV cross-linker (UVP, Inc., Upland, CA).

### Immunoprecipitation and western blot

For the immunoprecipitation (IP) experiment, cells were collected and lysed in lysis buffer (300mM NaCl, 50mM Tris pH 8.0, 0.4%NP-40, 10mM MgCl_2_, and 2.5mM CaCl_2_) supplemented with protease inhibitors cocktail (Complete mini EDTA-free; Roche Diagnostics, Mannheim, Germany). After centrifugation, the supernatant was measured using the BCA protein assay reagent (Pierce, Rockford, IL). Then, 1 mg of total cell extracts was diluted to 1 ml of dilution buffer (50 mM Tris, pH 8.0, 0.4% NP-40). After a preclear step with protein G sepharose beads, cell lysates were incubated with appropriate antibodies overnight at 4°C and then with 50 μl of a 50% slurry of proteinG sepharose for 2 hours. Immunoprecipitates were then washed five times in washing buffer (50 mM Tris, pH 8.0, 150 mM NaCl, 0.4% NP-40, and 5 mM MgCl_2_) and analyzed by Western blot. The protein bands were visualized using an IRDye800-conjugated secondary antibody; the infrared fluorescence image was obtained using an Odyssey infrared imaging system (Li-CorBioscience).

### Glutathione S-transferase pull-down

Recombinant glutathione S-transferase (GST) or GST-DCUN1D3 were expressed in Escherichia coli and purified. In addition, the quantity and quality of recombinant proteins were assessed by SDS-PAGE. Then, 1 μg of GST fusion protein or GST was incubated with whole cell lysates prepared from HA-CAND1 or Flag-CUL1 transfected HeLa cells overnight at 4°C. After five washes, beads were resuspended in 2× SDS loading buffer and analyzed by SDS-PAGE followed by Western blot.

### Cell cycle analysis

To prepare cells for fluorescence-activated cell sorter (FACS) analysis, 10^5^−10^6^ HeLa cells were fixed in 70% ethanol overnight at 4°C. After washing with PBS, cells were incubated with RNaseA (0.5 mg/mL) at 37°C for 30 min (Sigma). Finally, the cells were stained with propidium iodide (PI) (50 μg/mL) and analyzed by fluorescence-activated cell sorting (FACS) on a FACSCalibur instrument (Bectin Dickinson, San Jose, CA). DNA content per cell was measured using the CellQuest Pro program.

### Real-time PCR

RNA was isolated from cell lysates by Trizol and cDNA was synthesized from 1μg RNA using SuperScript III (Invitrogen), per the manufacturer's protocol. qRT-PCR was carried out on the CFX96 ^TM^Real time system (Bio-Rad) using gene-specific primers designed with the Primer3 software and synthesized by AuGCT Technologies (Beijing, China). qRT-PCR data were analyzed using a relative quantification method and plotted as average fold change compared to the control. actin was used as an internal reference. P27 Primers used for qPCR are as followings, p27 fw: GAAATCTCTTCGGCCCGGTC, p27 rv: CACTTGCGCTGACTCGCTTC, Actin fw: ACGGCTCCGGCATGTGCAAA, Actin rv: TTCCCACCATCACACCCTGG. Three technical replicates were utilized in each assay, and all data shown was performed with at least three biological replicates.

### *In vitro* neddylation assay

Cell lysates were prepared with 0.5% NP40 lysis buffer (0.5% NP-40, 50mM Tris [pH 7.4], 150mM NaCl) containing 10 μg/ml aprotinin, 1mM benzamidine, 10μg/ml leupeptin, and 50mM NaF, then clarified and normalized. Approximately one 100mm plate of confluent HeLa cells lysed in a volume of 200-300ul was used per reaction. Reactions were incubated 1 hour at 30°C in neddylation buffer (50mM Tris[pH7.4], 5mM MgCl_2_, 10mM creatine phosphate, 50μg/ml creatine kinase, and 1mM DTT) with final concentrations of 30mM ATP. CUL1 complexes were immunoprecipitated from these reactions overnight at 4°C and then subjected to SDS-PAGE and Western blot analysis to assay for neddylated CUL1.

### Statistics analysis

The one-way analysis of variance (ANOVA) was performed to compare the significance of different treatment in Figure 2 and Supplementary Figure S3 using SPSS 19.0. *p* < 0.05 was thought to be a significant difference.

## SUPPLEMENTARY FIGURES AND TABLES




