# Supplementary material for: DCUN1D3 activates SCFSKP2 ubiquitin E3 ligase activity and cell cycle progression under UV damage
Source: Oncotarget. 2016 Aug 16;7(36):58483–91. doi: 10.18632/oncotarget.11302 (PMC5295445; doi:10.18632/oncotarget.11302)
Supplement: Supplementary file 1 [file oncotarget-07-58483-s001.pdf]

DCUN1D3 activates SCF^{SKP2} ubiquitin E3 ligase activity and cell cycle progression under UV damage

SUPPLEMENTARY FIGURES AND TABLE

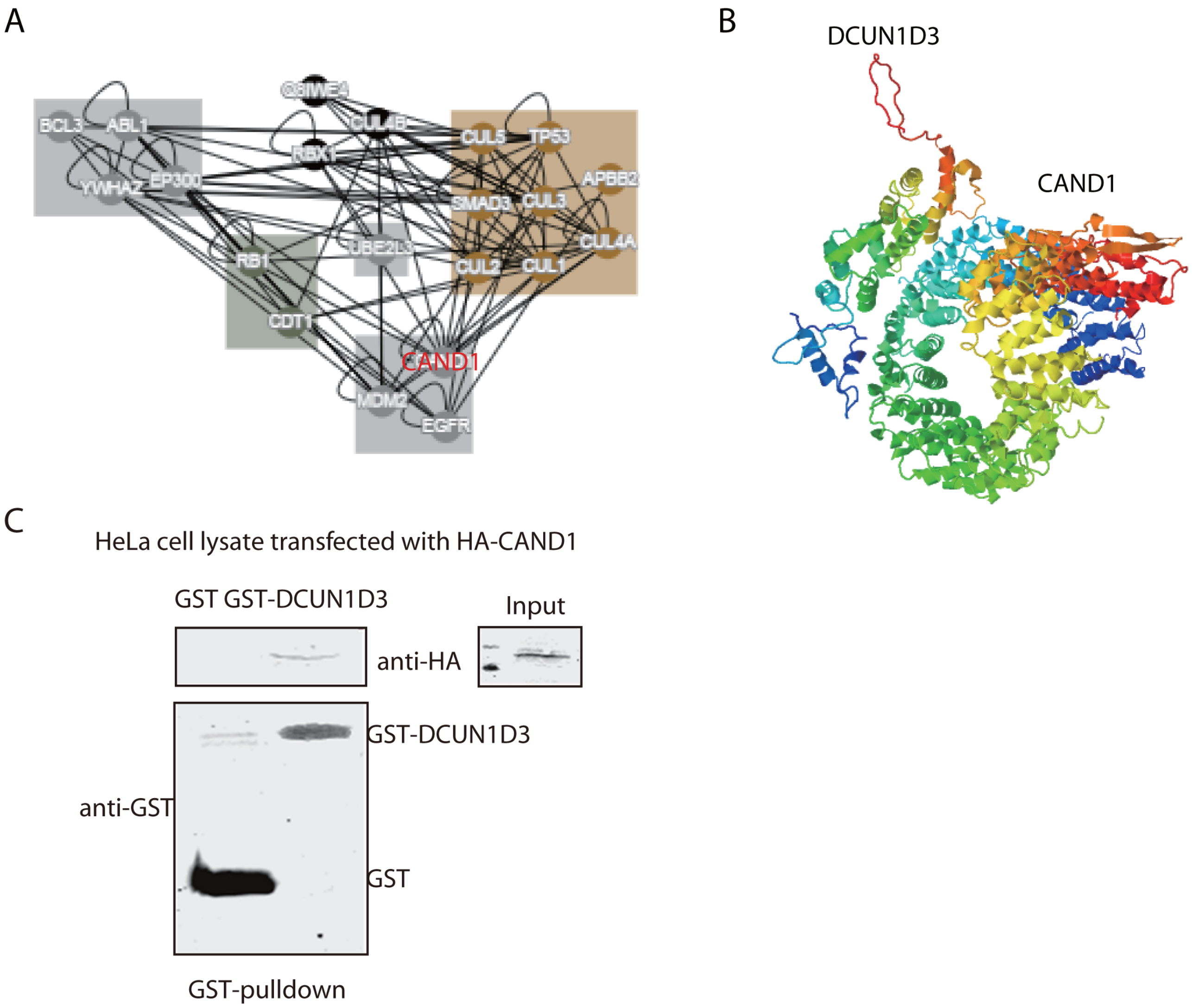

Supplementary Figure S1: A. An inteaction protein network generated from the PrePPI website. **B.** A DCUN1D3-CAND1 interaction model generated from the Frodock 2.0 (J.I. Garzón et al., 2009). **C.** Direct interaction of DCUN1D3 with CAND1 *in vitro*. The full-length GST-DCUN1D3 fusion protein and the GST protein were incubated with CAND1-transfected HeLa cell lysates overnight at 4°C. Washed beads were analyzed for the presence of CAND1 and GST by Western blot.

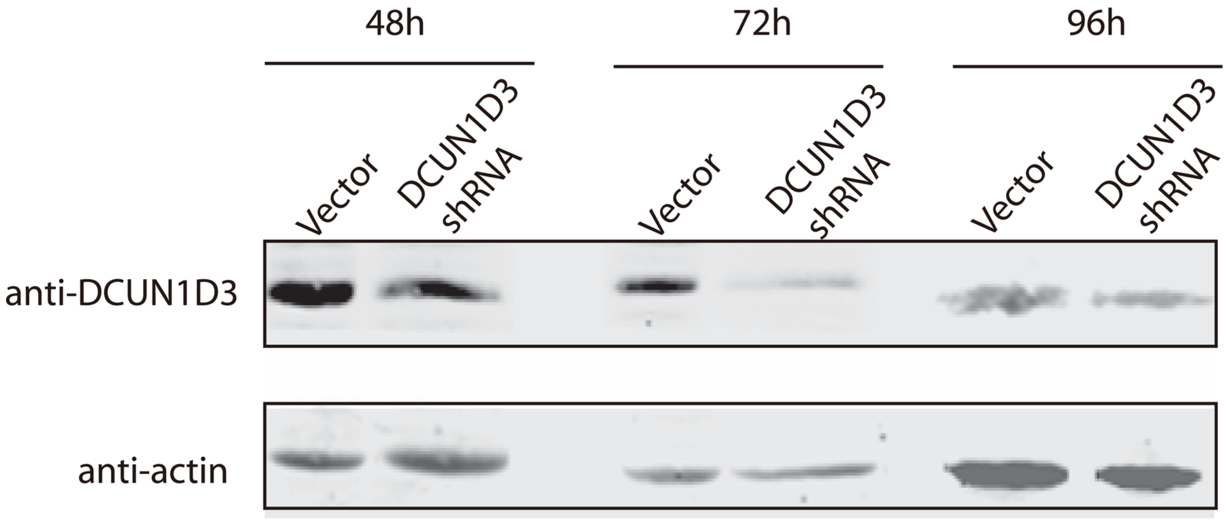

Supplementary Figure S2: Screening of effective DCUN1D3 shRNA. The DCUN1D3 shRNA and its control vectors were transfected into the HeLa cells. The shRNA effects were determined by Western blot at 48h, 72, 96h after UV irradiation.

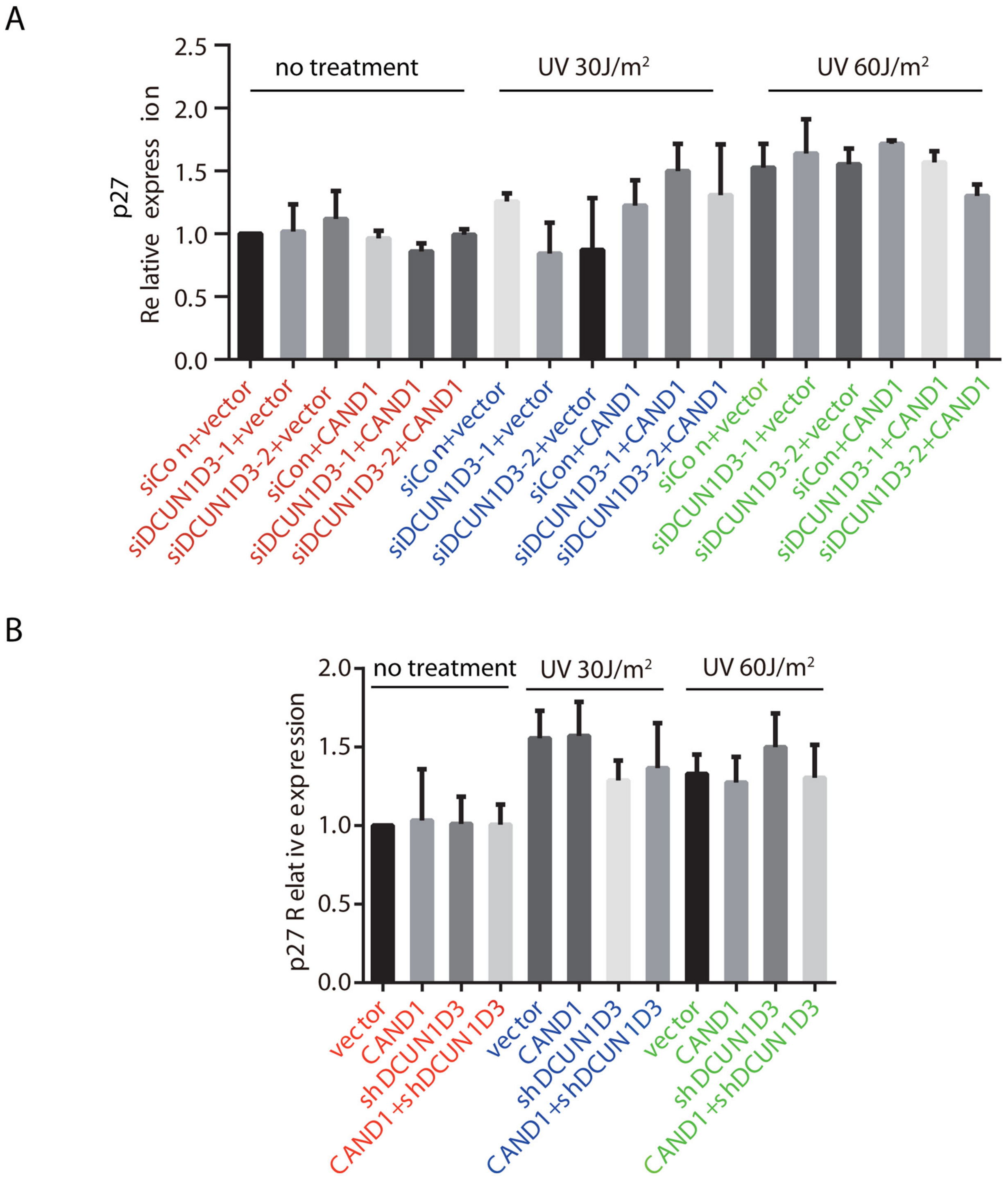

Supplementary Figure S3: Real-time PCR analysis of p27 mRNA level after various treatments. A. HeLa cells were transfected with DCUN1D3 siRNA combined either with vector or HA-CAND1, then treated with 30 J/m^2 UV or 60 J/m^2 UV. 24h after treatment, RNAs were collected and analyzed for RT-qPCR of p27. **B.** HeLa cells were transfected with HA-CAND1, DCUN1D3 shRNA solely or combined with HA-CAND1, then treated with 30 J/m^2 UV or 60 J/m^2 UV. 24h after treatment, cells were collected and analyzed for p27 expression.

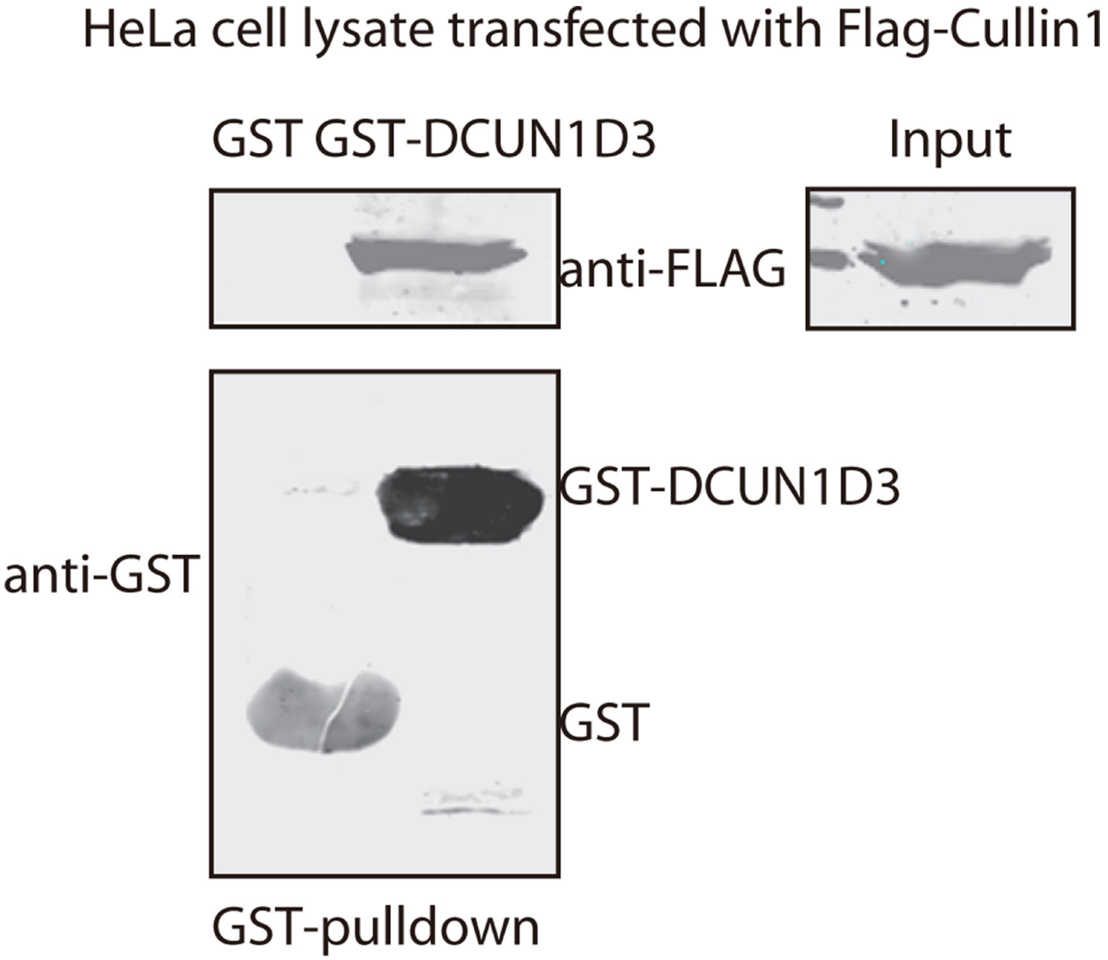

Supplementary Figure S4: Direct interaction of DCUN1D3 with CUL1 *in vitro*. The full-length GST-DCUN1D3 fusion protein and the GST protein were incubated with CUL1-transfected HeLa cell lysates overnight at 4°C. Washed beads were analyzed for the presence of CUL1 and GST by Western blot.

Supplementary Table S1: A complete list of predicted proteins that may interact with DCUN1D3

See Supplementary File 1
